# Crystal structure of binimetinib (Form A), C_17_H_15_BrF_2_N_4_O_3_, from synchrotron X-ray powder diffraction data and density functional theory

**DOI:** 10.1107/S2056989026004718

**Published:** 2026-05-15

**Authors:** Jean guillaume Ducreux, James A. Kaduk, Anja Dosen, Thomas N. Blanton

**Affiliations:** a Department of Chemistry North Central College, 131 S Loomis St Naperville IL 60540 USA; b ICDD, 12 Campus Blvd., Newtown Square PA, 19073, USA; University of Aberdeen, United Kingdom

**Keywords:** powder diffraction, binimetinib, Mektovi, Rietveld refinement, density functional theory

## Abstract

The crystal structure of binimetinib Form A [systematic name: 6-(4-bromo-2-fluoroanilino)-7-fluoro-*N*-(2-hydroxyethoxy)-3-methylbenzimidazole-5-carboxamide], C_17_H_15_BrF_2_N_4_O_3_, has been solved and refined using synchrotron X-ray powder diffraction data, and optimized using density functional theory techniques. It crystallizes in space group *P*2_1_2_1_2_1_ with one mol­ecule in the asymmetric unit at 298 K. The crystal structure consists of layers of mol­ecules lying parallel to the *bc* plane. Hydrogen bonds (O/N—H⋯N/O) link the layers *via* sheets in the *ac* plane. The commercial sample contains minor impurities of KH_2_(PO_4_) and Fe_0.33_Zr_2_(PO_4_)_3_.

## Abstract 2

The crystal structure of binimetinib (Form A) has been solved and refined using synchrotron X-ray powder diffraction data, and optimized using density functional theory techniques

## 1. Chemical context

Binimetinib, C_17_H_15_BrF_2_N_4_O_3_, sold under the brand name Mektovi, is an anti-cancer medication (Tran & Cohen, 2020 ▸). Administered orally, binimetinib is combined with encorafenib for the treatment of melanoma. Its systematic name (CAS Registry Number 606143-89-9) is 6-(4-bromo-2-fluoro­anilino)-7-fluoro-*N*-(2-hy­droxy­eth­oxy)-3-methyl­benzimidaz­ole-5-carboxamide.
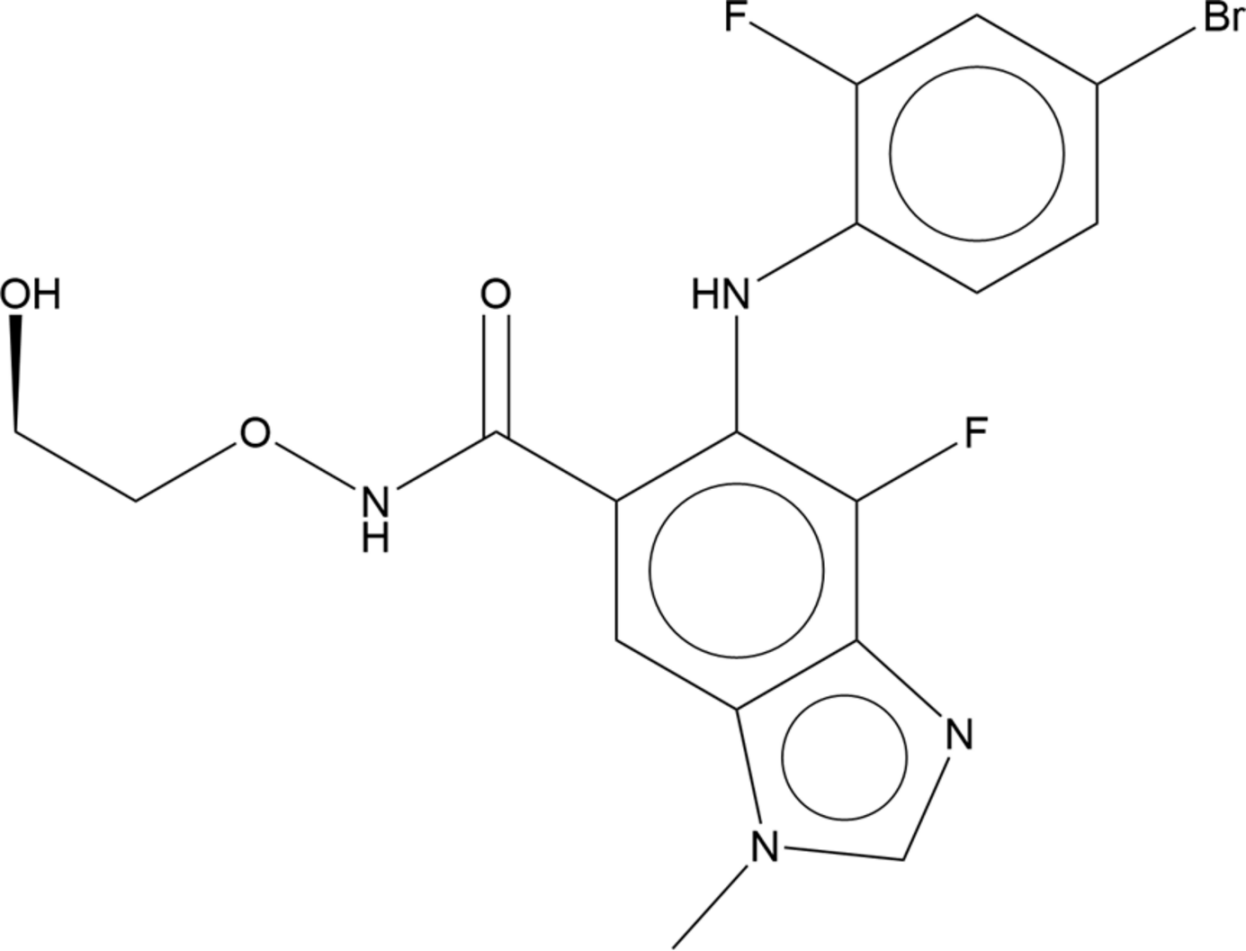


This work was carried out as part of a project (Kaduk *et al.*, 2014 ▸) to determine the crystal structures of large-volume commercial pharmaceuticals, and include high-quality powder diffraction data for them in the Powder Diffraction File (Kabekkodu *et al.*, 2024 ▸).

## 2. Structural commentary

**Figure 1:**
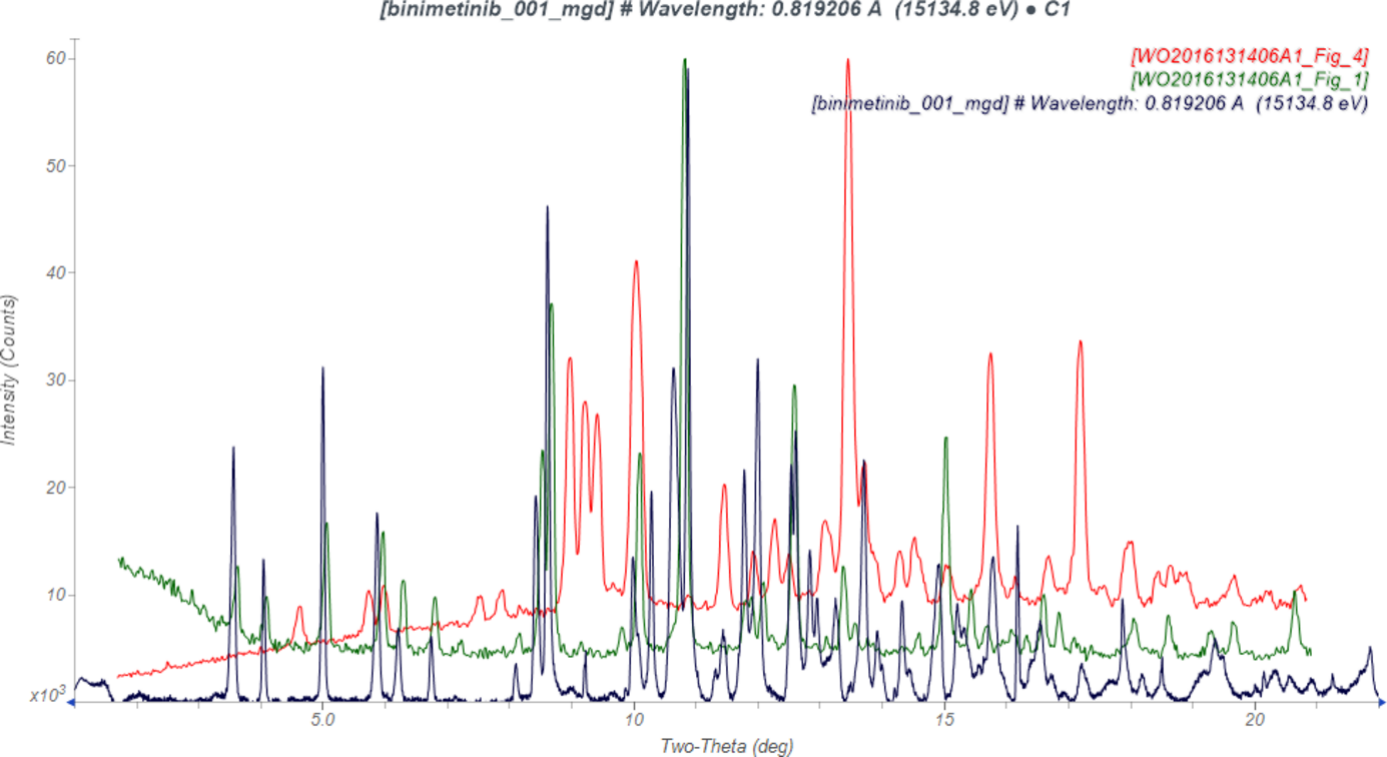
Comparison of the background-subtracted synchrotron pattern of binimetinib (black) to those for Form A (green) and Form B (red) reported by Chen *et al.* (2016 ▸). The patent patterns (measured using Cu *K*α radiation) were digitized using *UN-SCAN-IT* (Silk Scientific, 2013 ▸) and converted to the synchrotron wavelength of 0.819325 (2) Å using *JADE Pro* (MDI, 2025 ▸). Image generated using *JADE Pro* (MDI, 2025 ▸).

The powder pattern obtained in this study is similar enough to the one reported for binimetinib Form A by Chen *et al.* (2016 ▸) to conclude that they are the same material (Fig. 1 ▸). There are extra peaks in the synchrotron pattern indicating the presence of at least two crystalline impurities, which we have identified as 2.6% KH_2_(PO_4_) and approximately 0.3% Fe_0.33_Zr_2_(PO_4_)_3_. It would be inter­esting to understand how these phases came to be present in this commercial sample.

**Figure 2:**
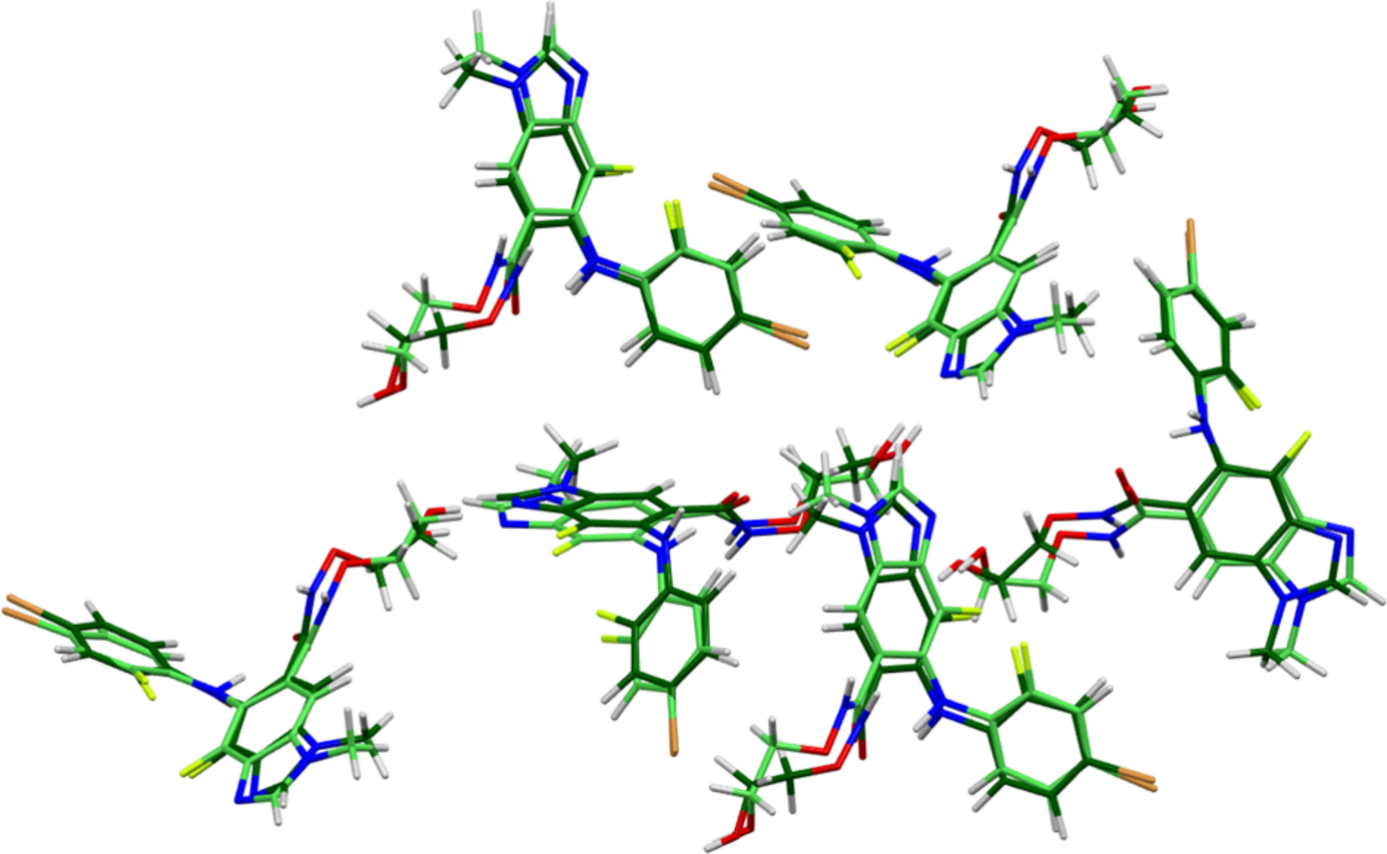
Comparison of the Rietveld-refined (colored by atom type) and *VASP*-optimized (light green) structures of binimetinib using the *Mercury* CSD-Materials/Search/Crystal Packing Similarity tool. The root-mean-square Cartesian displacement is 0.549 Å. Image generated using *Mercury* (Macrae *et al.*, 2020 ▸).

**Figure 3:**
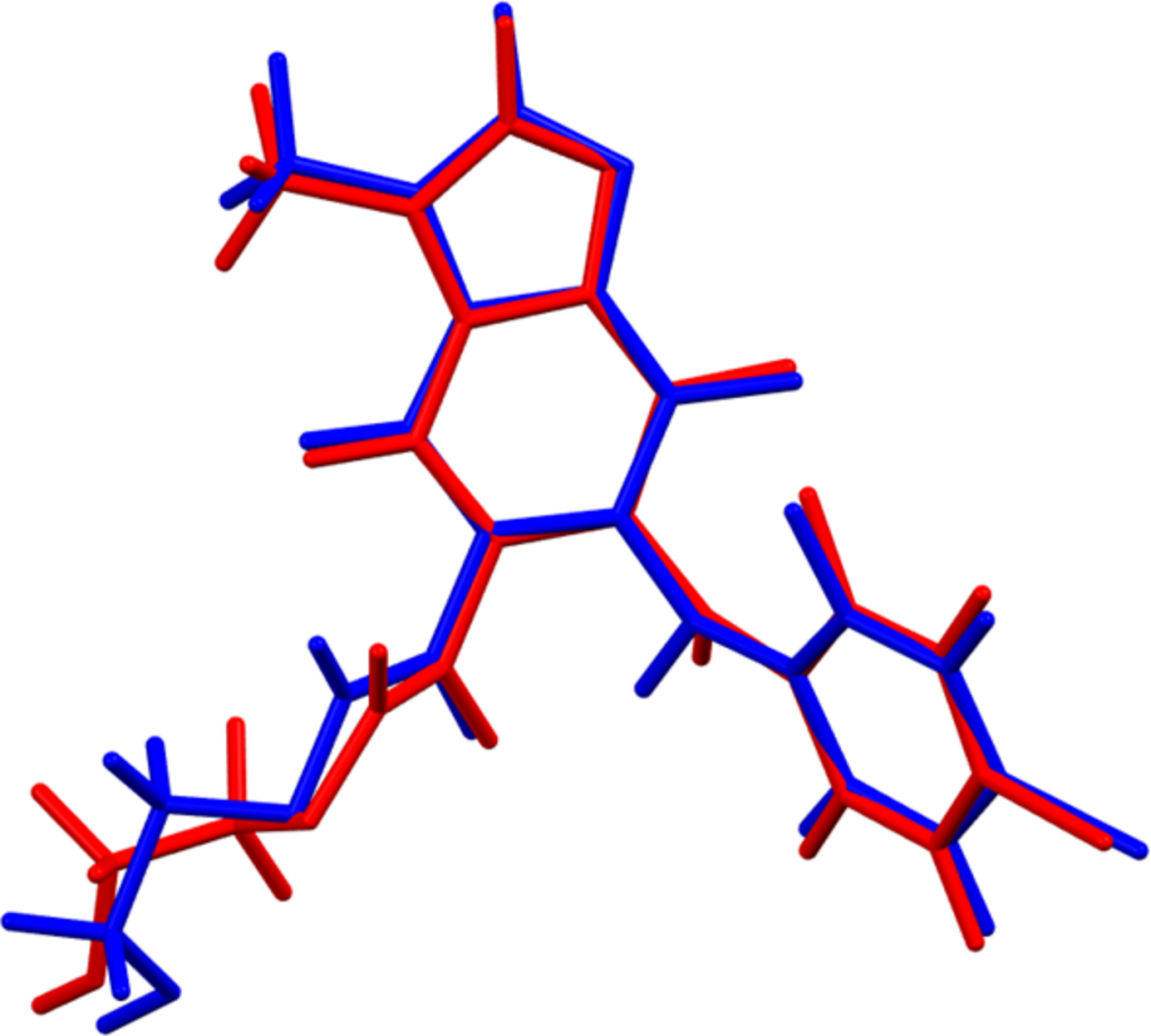
Comparison of the refined structure of binimetinib (red) to the *VASP*-optimized structure (blue). The comparison was generated using the *Mercury* Calculate/Mol­ecule Overlay tool; the r.m.s. difference is 0.392 Å. Image generated using *Mercury* (Macrae *et al.*, 2020 ▸).

**Figure 4:**
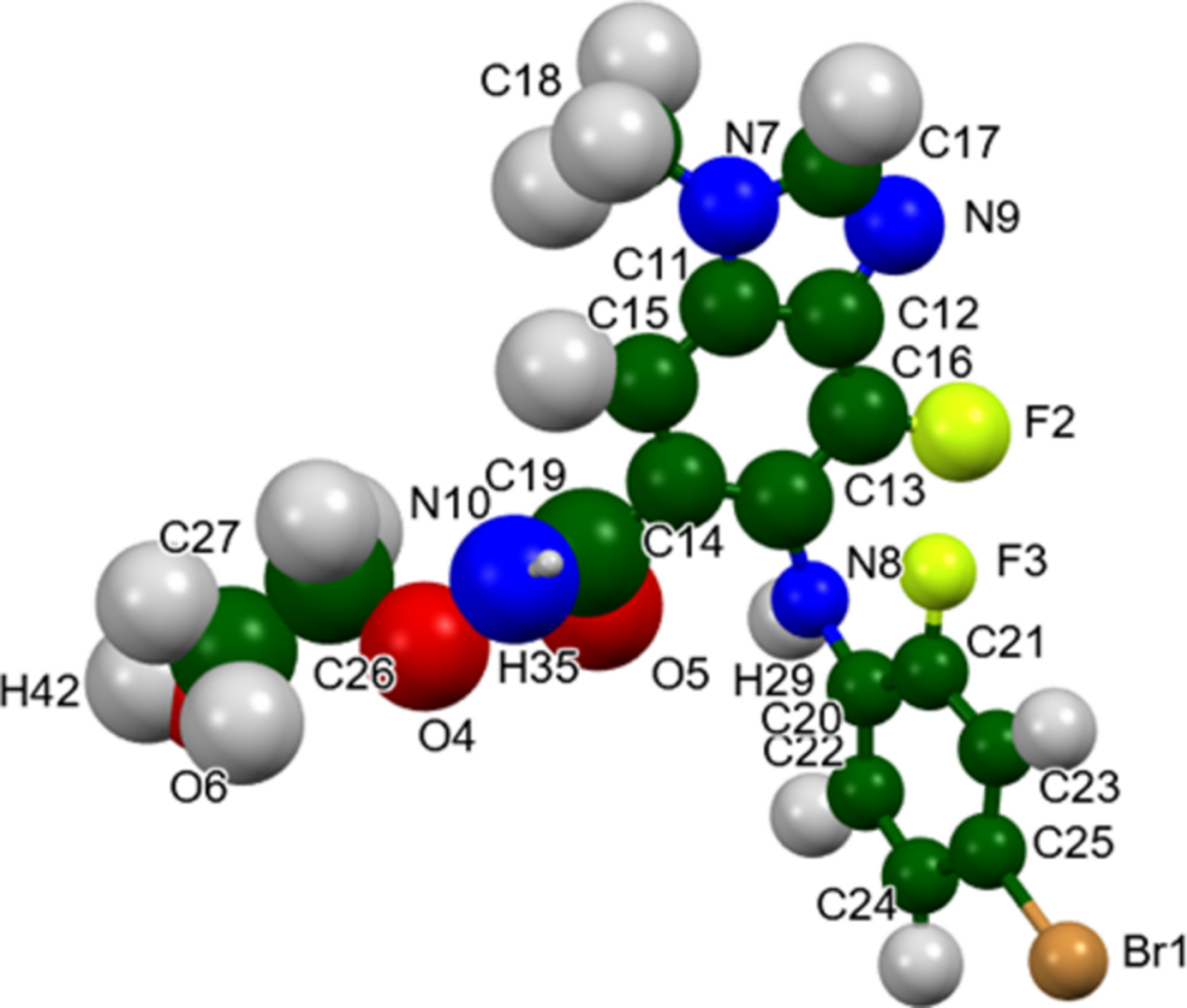
The asymmetric unit of binimetinib, with the atom numbering. The atoms are represented by 50% probability spheroids. Image generated using *Mercury* (Macrae *et al.*, 2020 ▸).

The root-mean-square difference of the non-H atoms in the Rietveld-refined and *VASP*-optimized structures of binimetinib, calculated using the *Mercury* CSD-Materials/Search/Crystal Packing similarity tool (Macrae *et al.*, 2020 ▸) is 0.549 Å (Fig. 2 ▸). The root-mean-square Cartesian displacement of the non-H atoms in the refined and optimized structures, calculated using the *Mercury* Calculate/Mol­ecule overlay tool, is 0.392 Å (Fig. 3 ▸). The largest differences are in the side chains. The agreements are just outside the normal range for correct structures (van de Streek & Neumann, 2014 ▸). Since the specimen was almost certainly changing due to exposure to the synchrotron beam, the accuracy of this structure might be lower than usual. The asymmetric unit is illustrated in Fig. 4 ▸. The remaining discussion will emphasize the *VASP*-optimized structure.

All of the bond distances, and most of the bond angles and torsion angles fall within the normal ranges indicated by a *Mercury* Mogul geometry check (Macrae *et al.*, 2020 ▸). The C21—C20—N8 bond angle [126.1°; average = 118.9 (22)°; *Z*-score = 3.2] is flagged as unusual. Torsion angles involving rotation about the N8—C20 bond are flagged as unusual. They lie on long tails of the distributions of similar torsion angles, so are unusual but not unprecedented. The O4—C26—C27—O5 torsion angle of −62.9° indicates a *gauche* conformation for the hy­droxy­ethyl side chain and the dihedral angle between the benzimidazole and phenyl ring mean planes is 57.0°.

Quantum chemical geometry optimization of the isolated binimetinib mol­ecule (DFT/B3LYP/6-31G*/water) using *Spartan ’24* (Wavefunction, 2025 ▸) indicated that the observed conformation is 6.2 kcal mol^−1^ higher in energy than the local minimum. The root-mean-square difference is 0.410 Å, and the maximum differences are in the amide group. The global minimum-energy conformation has a similar energy, but is much more compact (severely folded on itself). The mol­ecule is apparently flexible, and inter­molecular inter­actions are important in determining the solid-state conformation.

## 3. Supra­molecular features

**Figure 5:**
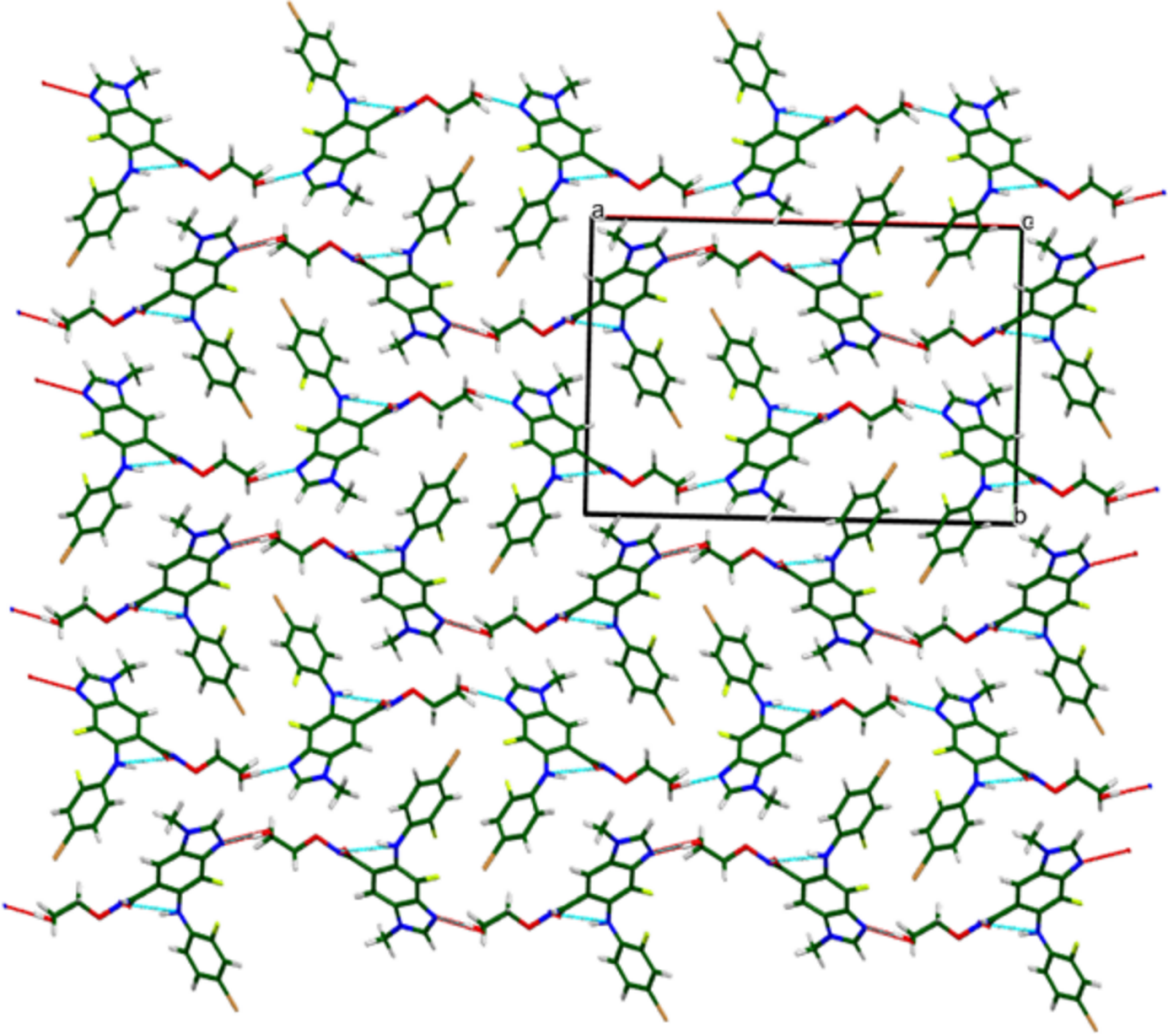
Crystal structure of binimetinib, viewed down the *c* axis. Image generated using *Mercury* (Macrae *et al.*, 2020 ▸).

The extended structure (Fig. 5 ▸) consists of layers lying parallel to the *bc* plane. Hydrogen bonds (O/N—H⋯N/O) link the layers *via* sheets in the *ac* plane. Analysis of the contributions to the total crystal energy of the structure using the Forcite module of Materials Studio (Dassault Systèmes, 2024 ▸) indicated that the intra­molecular energy is dominated by angle distortion terms. The inter­molecular energy is dominated by van der Waals attractions, which in this force field based analysis include hydrogen bonds. The hydrogen bonds are better discussed using the results of the DFT calculation.

Hydrogen bonds (Table 1 ▸) are prominent in the structure. The O6—H42⋯N9, N10—H35⋯O5, and N8—H29⋯O5 hydrogen bonds link the mol­ecules into sheets lying parallel to the *ac* plane. The energies of the N—H⋯O bonds were calculated using the correlation of Wheatley & Kaduk (2019 ▸). The O6—H42⋯N9 bond generates a graph-set descriptor (Etter, 1990 ▸; Bernstein *et al.*, 1995 ▸; Motherwell *et al.*, 2000 ▸) 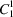
(12) and the N10—H35⋯O5 bond corresponds to graph set 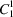
(4). Several C—H⋯O hydrogen bonds also contribute to the cohesion of the structure. By the Mulliken overlap population criterion, the C17—H30⋯O6 bond is exceptionally strong.

**Figure 6:**
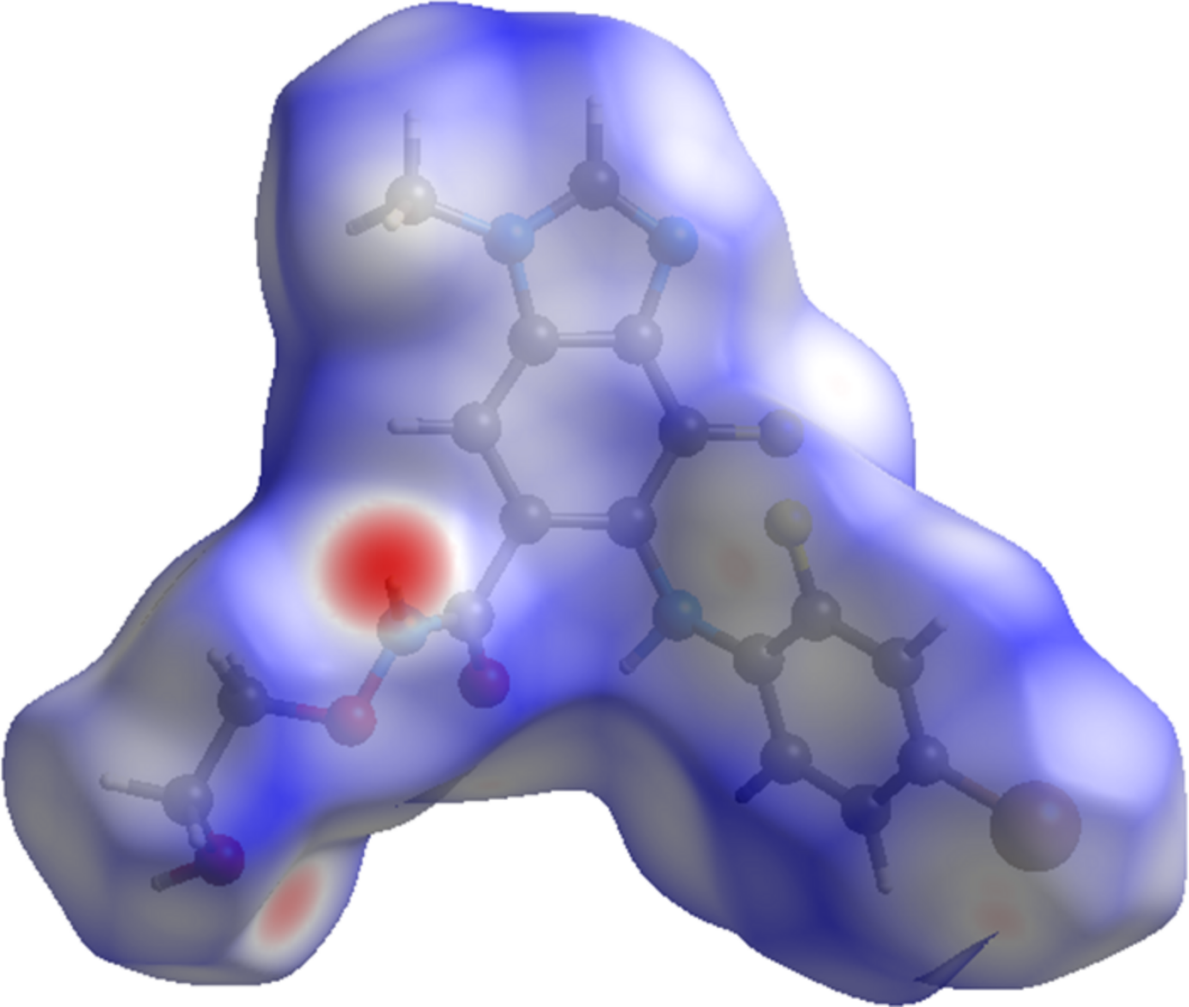
The Hirshfeld surface of binimetinib. Inter­molecular contacts longer than the sums of the van der Waals radii are colored blue, and contacts shorter than the sums of the radii are colored red. Contacts equal to the sums of radii are white. Image generated using *CrystalExplorer* (Spackman *et al.*, 2021 ▸).

The volume enclosed by the Hirshfeld surface of binimetinib (Fig. 6 ▸; Hirshfeld, 1977 ▸; Spackman *et al.*, 2021 ▸) is 443.0 Å^3^ or 98.1% of 1/4 of the unit-cell volume. The only significant close contacts (red in Fig. 6 ▸) involve the hydrogen bonds. The volume/non-hydrogen atom is smaller than usual, at 16.7 Å^3^, so the packing seems relatively dense.

**Figure 7:**
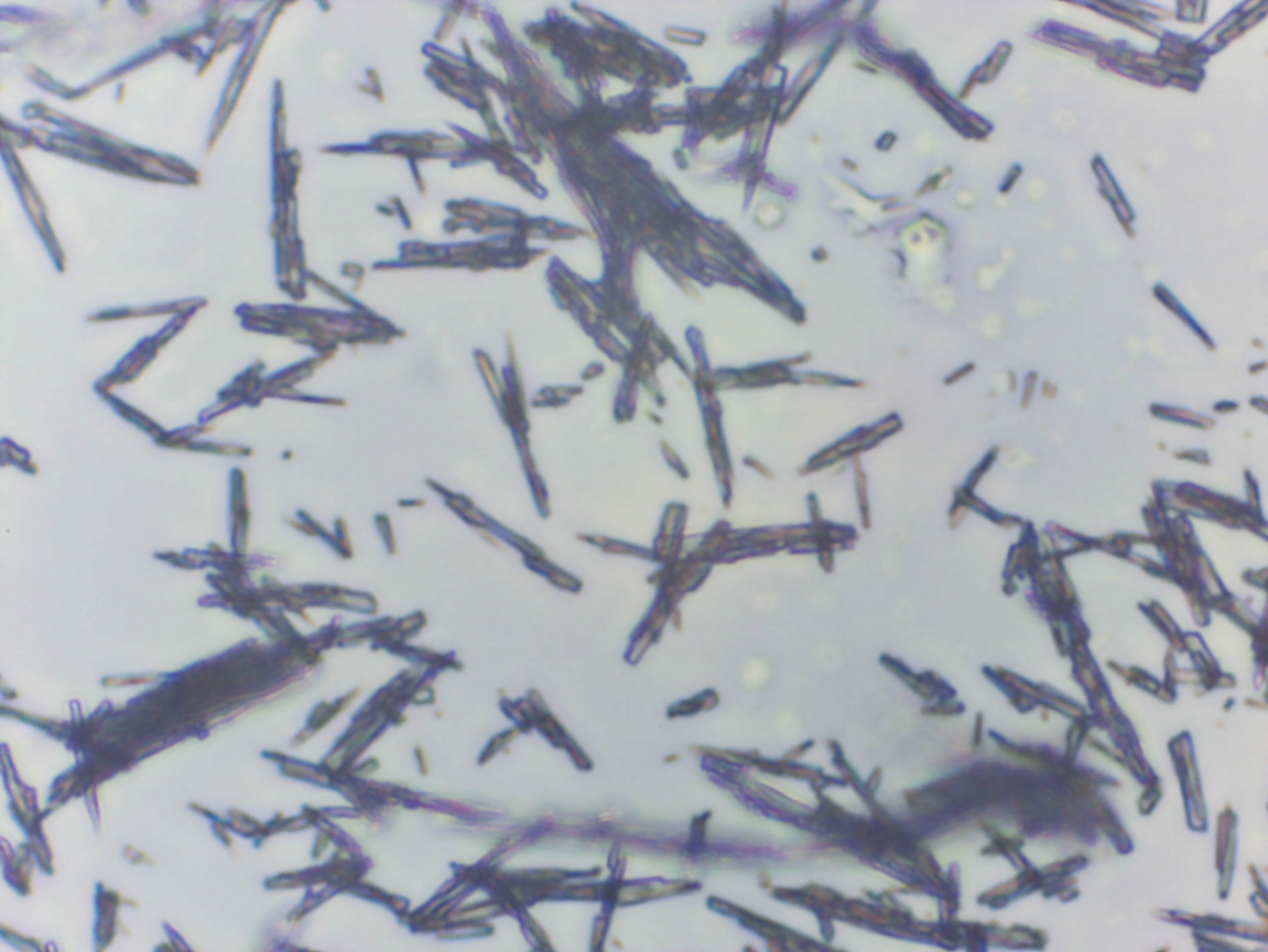
Optical micrograph of binimetinib. Original magnification = 40×.

The Bravais–Friedel–Donnay–Harker (Bravais, 1866 ▸; Friedel, 1907 ▸; Donnay & Harker, 1937 ▸) algorithm suggests that we might expect needle morphology for binimetinib, with [001] as the long axis, as expected from the anisotropy of the lattice parameters. A photomicrograph (Fig. 7 ▸) indicates needle morphology. A 2nd-order spherical harmonic model was included for preferred orientation. The texture index was 1.048 (3), indicating that preferred orientation was significant in this rotated capillary specimen.

## 4. Database survey

Powder patterns for crystalline Forms A and B of binimetinib are reported in Inter­national Patent Application WO 2016/131406 A1 (Chen *et al.*, 2016 ▸; Crystal Pharmatech Co. Ltd.), but no crystal structures were reported. The crystal structure of a DMSO adduct of binimetinib has been determined (Buist *et al.*, 2021 ▸; Johnson Matthey Public Limited Company), and X-ray powder diffraction data are reported. Powder data are also reported for a citric acid adduct. A Raman spectrum for binimetinib free base is reported, but no diffraction data. Amorphous binimetinib is claimed in Inter­national Patent Application WO 2021/116901 A1 (Palle *et al.*, 2021 ▸; Biocon Ltd.). A reduced-cell search in the Cambridge Structural Database ( CSD Conquest Build 2026.1.0; Groom *et al.*, 2016 ▸) yielded 15 hits for unrelated structures, but no structures for binimetinib or its derivatives.

## 5. Synthesis and crystallization

Binimetinib is a commercial reagent, purchased from TargetMol (Batch #146079), and was used as-received.

## 6. Refinement

Crystal data, data collection and structure refinement details are summarized in Table 2 ▸. The white powder was packed into a 0.5 mm diameter Kapton capillary, and rotated during the measurement at ∼2 Hz. The powder pattern was measured at 298 (1) K at the Wiggler Low Energy Beamline (Leontowich *et al.*, 2021 ▸) of the Brockhouse X-ray Diffraction and Scattering Sector of the Canadian Light Source using a wavelength of 0.819325 (2) Å (15.1 keV) from 1.6–75.0° 2θ with a step size of 0.0025° and a collection time of 3 minutes. The high-resolution powder diffraction data were collected using eight Dectris Mythen2 X series 1K linear strip detectors. NIST SRM 660b LaB_6_ was used to calibrate the instrument and refine the monochromatic wavelength used in the experiment.

Illuminating a Br-containing specimen with 15 keV X-rays results in severe fluorescent background (and thus eventually low residuals). It would be very surprising if this sample did not exhibit beam damage, as C—Br bonds are known to be prone to photolysis.

The pattern was difficult to index. Visual examination of the raw data indicated several very sharp peaks at high angles, probably indicating an inorganic impurity. Initial indexing using *N-TREOR* (Altomare *et al.*, 2013 ▸) yielded a *P*2_1_/*c* cell with *a* = 5.36109, *b* = 23.26552, *c* = 16.20200 Å, *β* = 95.864°, *V* = 2010.3 Å^3^, and *Z* = 4. Although the structure could be solved and refined using this cell, the structure was unsatisfactory - both for the residuals (*R*_wp_ = 0.0267) and the agreement of observed and calculated peak positions, even considering potential beam damage.

Indexing with *DICVOL14* (Louër & Boultif, 2014 ▸), permitting up to three unindexed peaks, yielded a primitive monoclinic cell with *a* = 23.2656, *b* = 16.0153, *c* = 4.8916 Å, *β* = 90.420°, *V* = 1822.57 Å^3^, and *Z* = 4. The *β* angle being close to 90° suggested that we consider the possibility that the cell was ortho­rhom­bic. The space-group-inter­pretation routine of *EXPO2014* (Altomare *et al.*, 2013 ▸) suggested space group *P*2_1_2_1_2_1_, which was confirmed by successful solution and refinement of the structure.

The mol­ecular structure of binimetinib was downloaded from PubChem (Kim *et al.*, 2023 ▸) as Conformer3D_COMPOUND_CID_10288191.sdf. It was converted to a *.mol2 file using *Mercury* (Macrae *et al.*, 2020 ▸), and to a Fenske–Hall *Z*-matrix using *OpenBabel* (O’Boyle *et al.*, 2011 ▸). The crystal structure was solved by Monte Carlo simulated annealing techniques as implemented in *EXPO2014* (Altomare *et al.*, 2013 ▸) using the binimetinib mol­ecule as the fragment, including a bump penalty on the non-H atoms and (001) preferred orientation. For the structure solution, a constant 49,000 counts were subtracted from the raw data, to minimize the effect of the high background. The structure was also solved using parallel tempering techniques as implemented in *FOX* (Favre-Nicolin & Černý, 2002 ▸), including (001) preferred orientation. Essentially the same structure was obtained from the two programs, and the *FOX* solution was adopted for refinement.

Indexing the sharp high-angle peaks using *DICVOL14* yielded a primitive tetra­gonal cell with *a* = 7.4557, *c* = 6.9776 Å, and *V* = 387.87 Å^3^. A reduced cell search in the Powder Diffraction File yielded several metallic phases, as well as (ortho­rhom­bic) KH_2_PO_4_ (PDF entry 04-016-0040; Baur, 1973 ▸). Including this phase in the refinement revealed the presence of additional peaks, which are best accounted for by a zirconium phosphate phase such as Fe_0.33_Zr_2_(PO_4_)_3_ (PDF entry 04-015-1781; Gobechiya *et al.*, 2004 ▸), which was included as a third phase.

Rietveld refinement was carried out with *GSAS-II* (Toby & Von Dreele, 2013 ▸). Only the 3.3–35.0° portion of the pattern was included in the refinements (*d_min_* = 1.362 Å). All non-H bond distances and angles were subjected to restraints, based on a *Mercury* Mogul Geometry Check (Sykes *et al.*, 2011 ▸; Bruno *et al.*, 2004 ▸). The Mogul average and standard deviation for each qu­antity were used as the restraint parameters. The aromatic rings were restrained to be planar. The restraints contributed 7.5% to the overall *χ^2^*. The hydrogen atoms were included in calculated positions, which were recalculated during the refinement using *Materials Studio* (Dassault Systèmes, 2024 ▸). The *U_iso_* were grouped by chemical similarity. An attempt to refine the Br atom anisotropically yielded a non-positive-definite ellipsoid, and so was abandoned. The peak profiles were described using the generalized microstrain model (Stephens, 1999 ▸). The background was modeled using a six-term shifted Chebyshev polynomial, with a peak at 11.21° to model the scattering from the Kapton capillary and any amorphous component of the sample.

**Figure 8:**
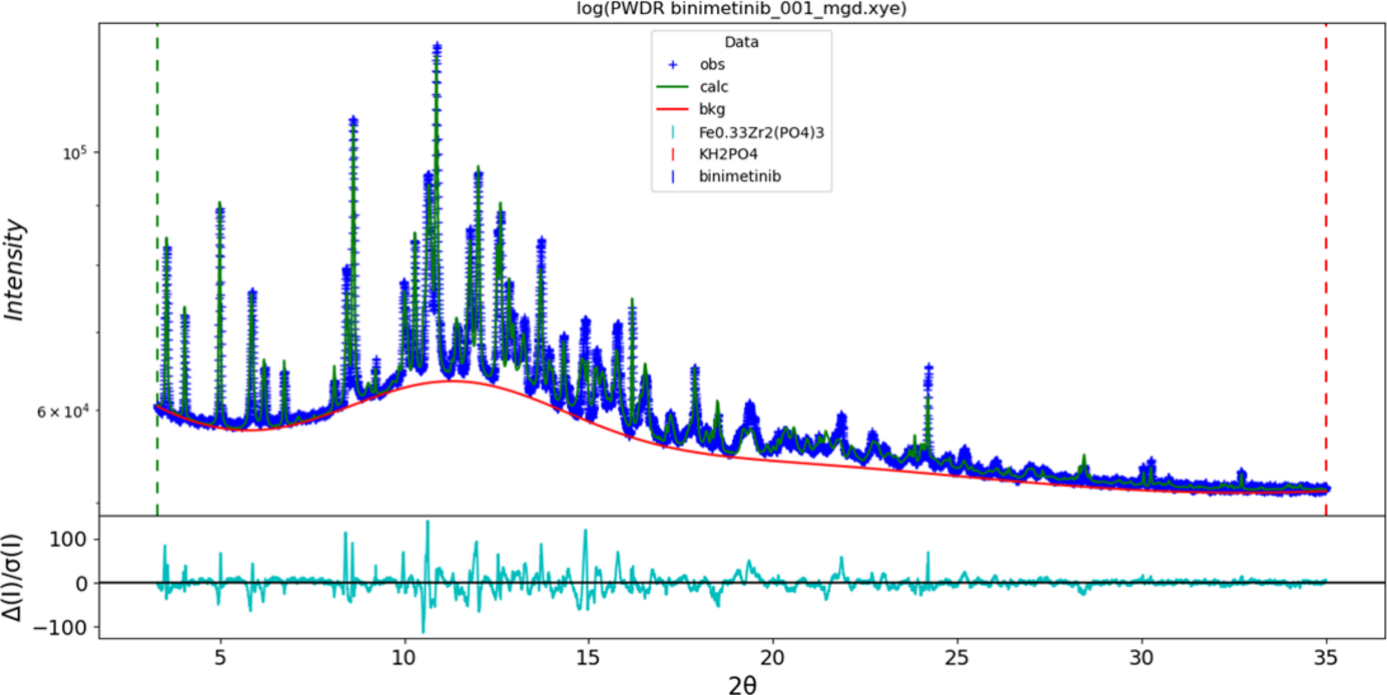
The Rietveld plot for binimetinib. The blue crosses represent the observed data points, and the green line is the calculated pattern. The cyan curve is the normalized error plot, and the red line is the background curve. The vertical scale is logarithmic.

The final refinement of 113 variables using 12,681 observations and 72 restraints yielded the residual *R_wp_* = 0.01891. The largest peak (1.62 Å from F2) and hole (1.92 Å from O6) in the difference-Fourier map are 0.33 (9) and −0.31 (9) *e* Å^−3^, respectively. The final Rietveld plot is shown in Fig. 8 ▸. The largest features in the normalized error plot are in the positions of some of the strong low-angle peaks, and probably indicate beam damage.

The crystal structure of binimetinib was optimized (fixed experimental unit cell) with density functional theory techniques using *VASP* (Kresse & Furthmüller, 1996 ▸) through the *MedeA* graphical inter­face (Materials Design, 2024 ▸). The calculation was carried out on 32 cores of a 144-core (768 Gb memory) HPE Superdome Flex 280 Linux server at North Central College. The calculation used the GGA-PBE functional, a plane wave cutoff energy of 400.0 eV, and a *k*-point spacing of 0.5 Å^−1^ leading to a 3 × 3 × 1 mesh, and took ∼7.4 h. Single-point density functional theory calculations and population analysis were carried out using *CRYSTAL23* (Erba *et al.*, 2023 ▸) (fixed experimental cell) and population analysis was carried out using *CRYSTAL17* (Dovesi *et al.*, 2018 ▸), using a fixed experimental cell. The basis sets for the H, C, N and O atoms in the calculation were those of Gatti *et al.* (1994 ▸), and those for Br and F were from Peintinger *et al.* (2013 ▸). The calculations were run on a 3.5 GHz PC using 8 *k*-points and the B3LYP functional, and took ∼2.1 hr

## Supplementary Material

Crystal structure: contains datablock(s) binimetinib_3_publ, binimetinib_3_overall, binimetinib_3_phase_2, binimetinib_3_phase_1, binimetinib_3_phase_0, binimetinib_3_pwd_0, binimetinib_VASP. DOI: 10.1107/S2056989026004718/hb8206sup1.cif

CCDC references: 2552042, 2552043, 2552044, 2552045

Additional supporting information:  crystallographic information; 3D view; checkCIF report

## Figures and Tables

**Table 1:** Hydrogen-bond geometry (Å, °)

*D*—H⋯*A*	*D*—H	H⋯*A*	*D*⋯*A*	*D*—H⋯*A*
N8—H29⋯O5	1.02	1.90	2.727	136
N10—H35⋯O5^i^	1.04	1.67	2.675	161
O6—H42⋯N9^ii^	1.00	1.81	2.806	176
C17—H30⋯O6^iii^	1.09	2.18	3.221	158
C18—H32⋯O5^iii^	1.10	2.53	3.288	125
C26—H38⋯Br1^iv^	1.10	2.92	3.841	142
C26—H39⋯Br1^iii^	1.10	2.78	3.486	122

Symmetry codes: (i) 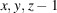; (ii) 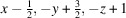; (iii) 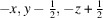; (iv) 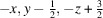.

**Table 2:** Experimental details

Crystal data	
Chemical formula	C_17_H_15_BrF_2_N_4_O_3_
*M*_r_	441.23
Crystal system, space group	Orthorhombic, *P*2_1_2_1_2_1_
Temperature (K)	298
*a*, *b*, *c* (Å)	23.1607 (11), 16.0073 (11), 4.86578 (17)
*V* (Å^3^)	1803.9 (2)
*Z*	4
Radiation type	Synchrotron, λ = 0.81933 Å
Specimen shape, size (mm)	Cylinder, 0.45 × 0.15

Data collection	
Diffractometer	Wiggler Low Energy Beamline, Brockhouse X-ray Diffraction and Scattering Sector, Canadian Light Source
Specimen mounting	Kapton capillary
Data collection mode	Transmission
Scan method	Step
2θ values (°)	2θ_min_ = −9.008, 2θ_max_ = 75.047, 2θ_step_ = 0.003

Refinement	
*R* factors and goodness of fit	*R*_p_ = 0.011, *R*_wp_ = 0.018, *R*_exp_ = 0.001, χ^2^ = 347.375
No. of parameters	113
No. of restraints	72
(Δ/σ)_max_	3.163

Computer programs: *GSAS-II* (Toby & Von Dreele, 2013 ▸).

## supplementary crystallographic information

### (binimetinib_3_phase_2) Crystal data

**Table:**

FeO_22_P_3_Zr_8_	β = 125.16°
*M**_r_* = 1230.51	*V* = 980.73 Å^3^
Monoclinic, *P*2_1_/*c*	*Z* = 4
*a* = 8.8395 Å	*D*_x_ = 8.334 Mg m^−^^3^
*b* = 8.9359 Å	*T* = 298 K
*c* = 15.1869 Å	

### (binimetinib_3_phase_2) Refinement

**Table:**

Weighting scheme based on measured s.u.'s	Preferred orientation correction: March-Dollase correction coef. = 1.000 axis = [0, 0, 1]

### (binimetinib_3_phase_2) Fractional atomic coordinates and isotropic or equivalent isotropic displacement parameters (Å^2^)

**Table:**

	*x*	*y*	*z*	*U*_iso_*/*U*_eq_	
Fe1	0.54200	0.20100	−0.17100	0.0139*	
Zr2	0.13090	0.02910	−0.11940	0.0101*	
Zr3	0.35780	0.03930	−0.39020	0.0127*	
P4	−0.03800	0.25400	−0.50400	0.0127*	
P5	0.24800	0.38200	−0.14700	0.0114*	
P6	0.53100	0.39800	−0.35400	0.0127*	
O7	−0.01200	0.32800	−0.58200	0.0165*	
O8	−0.22200	0.17400	−0.57300	0.0165*	
O9	−0.04000	0.35500	−0.42700	0.0139*	
O10	0.12300	0.14400	−0.43200	0.0139*	
O11	0.20400	0.22000	−0.17000	0.0215*	
O12	0.30100	0.42400	−0.03500	0.0203*	
O13	0.40100	0.40900	−0.16100	0.0177*	
O14	0.08900	0.48300	−0.22700	0.0152*	
O15	0.49900	0.24300	−0.32900	0.0165*	
O16	0.69700	0.38900	−0.36000	0.0152*	
O17	0.35600	0.45100	−0.46100	0.0152*	
O18	0.56500	0.51900	−0.26800	0.0139*	

### (binimetinib_3_phase_1) Crystal data

**Table:**

H_2_KO_4_P	*c* = 6.9755 (3) Å
*M**_r_* = 136.08	*V* = 387.75 (4) Å^3^
Orthorhombic, *P*2_1_2_1_2_1_	*Z* = 4
*a* = 7.4487 (5) Å	*D*_x_ = 2.331 Mg m^−^^3^
*b* = 7.4626 (5) Å	*T* = 298 K

### (binimetinib_3_phase_1) Refinement

**Table:**

Weighting scheme based on measured s.u.'s	Preferred orientation correction: March-Dollase correction coef. = 1.000 axis = [0, 0, 1]

### (binimetinib_3_phase_1) Fractional atomic coordinates and isotropic or equivalent isotropic displacement parameters (Å^2^)

**Table:**

	*x*	*y*	*z*	*U*_iso_*/*U*_eq_	
P1	0.00000	0.25000	0.37500	0.0100*	
K2	0.00000	0.25000	0.87500	0.0100*	
O3	0.08970	0.40150	0.50060	0.0100*	
O4	0.15570	1.16810	1.24930	0.0100*	
O5	0.92620	1.10680	0.50530	0.0100*	
O6	0.86180	0.33310	1.24490	0.0100*	
H7	0.23200	0.39600	0.50300	0.0100*	
H8	0.14600	1.02600	1.25700	0.0100*	

### 6-(4-Bromo-2-fluoroanilino)-7-fluoro-N-(2-hydroxyethoxy)-3-methylbenzimidazole-5-carboxamide (binimetinib_3_phase_0) Crystal data

**Table:**

C_17_H_15_BrF_2_N_4_O_3_	*V* = 1803.9 (2) Å^3^
*M**_r_* = 441.23	*Z* = 4
Orthorhombic, *P*2_1_2_1_2_1_	*D*_x_ = 1.625 Mg m^−^^3^
*a* = 23.1607 (11) Å	Synchrotron radiation
*b* = 16.0073 (11) Å	*T* = 298 K
*c* = 4.86578 (17) Å	cylinder, 0.45 × 0.15 mm

### 6-(4-Bromo-2-fluoroanilino)-7-fluoro-N-(2-hydroxyethoxy)-3-methylbenzimidazole-5-carboxamide (binimetinib_3_phase_0) Data collection

**Table:**

Wiggler Low Energy Beamline, Brockhouse X-ray Diffraction and Scattering Sector, Canadian Light Source diffractometer	Data collection mode: transmission
Specimen mounting: Kapton capillary	Scan method: step

### 6-(4-Bromo-2-fluoroanilino)-7-fluoro-N-(2-hydroxyethoxy)-3-methylbenzimidazole-5-carboxamide (binimetinib_3_phase_0) Refinement

**Table:**

Weighting scheme based on measured s.u.'s	Preferred orientation correction: Simple spherical harmonic correction Order = 2 Coefficients: 0:0:C(2,0) = -0.416(16); 0:0:C(2,2) = -0.373(18)

### 6-(4-Bromo-2-fluoroanilino)-7-fluoro-N-(2-hydroxyethoxy)-3-methylbenzimidazole-5-carboxamide (binimetinib_3_phase_0) Fractional atomic coordinates and isotropic or equivalent isotropic displacement parameters (Å^2^)

**Table:**

	*x*	*y*	*z*	*U*_iso_*/*U*_eq_	
Br1	0.2140 (2)	1.1878 (4)	0.6685 (13)	0.102 (4)*	
F2	0.1638 (7)	0.7558 (12)	0.763 (4)	0.162 (7)*	
F3	0.1628 (6)	0.8853 (7)	0.467 (3)	0.097 (8)*	
O4	−0.1140 (6)	0.9111 (10)	0.360 (4)	0.271 (10)*	
O5	−0.0394 (9)	0.8638 (15)	0.772 (3)	0.271 (10)*	
O6	−0.2407 (14)	0.9150 (17)	0.579 (6)	0.271 (10)*	
N7	0.0781 (5)	0.6083 (9)	0.073 (3)	0.162 (7)*	
N8	0.0739 (7)	0.8714 (7)	0.828 (3)	0.097 (8)*	
N9	0.1533 (6)	0.6159 (12)	0.362 (4)	0.162 (7)*	
N10	−0.0612 (7)	0.8681 (15)	0.320 (3)	0.271 (10)*	
C11	0.0672 (5)	0.6757 (12)	0.243 (4)	0.162 (7)*	
C12	0.1152 (5)	0.6812 (9)	0.415 (3)	0.162 (7)*	
C13	0.0721 (5)	0.8048 (8)	0.629 (4)	0.162 (7)*	
C14	0.0224 (5)	0.7937 (9)	0.465 (4)	0.162 (7)*	
C15	0.0191 (6)	0.7269 (11)	0.278 (5)	0.162 (7)*	
C16	0.1161 (8)	0.7456 (16)	0.607 (6)	0.162 (7)*	
C17	0.1321 (6)	0.5817 (14)	0.138 (5)	0.162 (7)*	
C18	0.0347 (7)	0.5616 (15)	−0.082 (5)	0.162 (7)*	
C19	−0.0326 (6)	0.8372 (8)	0.537 (3)	0.271 (10)*	
C20	0.1037 (9)	0.9484 (8)	0.791 (5)	0.097 (8)*	
C21	0.1489 (5)	0.9534 (6)	0.606 (3)	0.097 (8)*	
C22	0.0928 (12)	1.0187 (11)	0.955 (7)	0.097 (8)*	
C23	0.1845 (9)	1.0204 (8)	0.584 (6)	0.097 (8)*	
C24	0.1232 (13)	1.0924 (10)	0.906 (7)	0.097 (8)*	
C25	0.1697 (10)	1.0907 (8)	0.726 (6)	0.097 (8)*	
C26	−0.1620 (8)	0.8543 (15)	0.313 (9)	0.271 (10)*	
C27	−0.2166 (9)	0.9025 (19)	0.313 (5)	0.271 (10)*	
H28	−0.02487	0.71766	0.16889	0.2456*	
H29	0.05232	0.86998	1.03075	0.1171*	
H30	0.15757	0.54516	−0.01714	0.2456*	
H31	−0.01029	0.58209	−0.01501	0.2456*	
H32	0.03773	0.49577	−0.02975	0.2456*	
H33	0.03549	0.57555	−0.30781	0.2456*	
H34	0.05590	1.02227	1.10109	0.1171*	
H35	−0.04360	0.86005	0.10945	0.3252*	
H36	0.22406	1.02131	0.43551	0.1171*	
H37	0.11483	1.15369	1.03039	0.1171*	
H38	−0.16330	0.80548	0.48347	0.3252*	
H39	−0.15623	0.82195	0.10608	0.3252*	
H40	−0.20864	0.96693	0.22067	0.3252*	
H41	−0.24952	0.86822	0.18029	0.3252*	
H42	0.22400	0.59610	0.45600	0.3252*	

### 6-(4-Bromo-2-fluoroanilino)-7-fluoro-N-(2-hydroxyethoxy)-3-methylbenzimidazole-5-carboxamide (binimetinib_3_phase_0) Geometric parameters (Å, º)

**Table:**

Br1—C25	1.883 (3)	C19—O5	1.231 (4)
F2—C16	1.347 (4)	C19—N10	1.343 (3)
F3—C21	1.322 (5)	C19—C14	1.492 (4)
O4—N10	1.417 (4)	C20—N8	1.422 (5)
O4—C26	1.454 (6)	C20—C21	1.386 (2)
O5—C19	1.231 (4)	C20—C22	1.403 (5)
O6—C27	1.421 (10)	C21—F3	1.322 (5)
O6—H42^i^	0.85 (3)	C21—C20	1.386 (2)
N7—C11	1.380 (3)	C21—C23	1.357 (3)
N7—C17	1.359 (4)	C22—C20	1.403 (5)
N7—C18	1.462 (3)	C22—C24	1.394 (7)
N8—C13	1.439 (5)	C22—H34	1.113 (7)
N8—C20	1.422 (5)	C23—C21	1.357 (3)
N8—H29	1.107 (11)	C23—C25	1.366 (4)
N9—C12	1.392 (3)	C23—H36	1.167 (7)
N9—C17	1.317 (3)	C24—C22	1.394 (7)
N10—O4	1.417 (4)	C24—C25	1.385 (4)
N10—C19	1.343 (3)	C24—H37	1.170 (9)
N10—H35	1.110 (15)	C25—Br1	1.883 (3)
C11—N7	1.380 (3)	C25—C23	1.366 (4)
C11—C12	1.395 (3)	C25—C24	1.385 (4)
C11—C15	1.395 (4)	C26—O4	1.454 (6)
C12—N9	1.392 (3)	C26—C27	1.481 (9)
C12—C11	1.395 (3)	C26—H38	1.14 (4)
C12—C16	1.393 (2)	C26—H39	1.14 (3)
C13—N8	1.439 (5)	C27—O6	1.421 (10)
C13—C14	1.413 (3)	C27—C26	1.481 (9)
C13—C16	1.397 (3)	C27—H40	1.14 (3)
C14—C13	1.413 (3)	C27—H41	1.14 (3)
C14—C15	1.405 (3)	H28—C15	1.159 (10)
C14—C19	1.492 (4)	H29—N8	1.107 (11)
C15—C11	1.395 (4)	H30—C17	1.121 (13)
C15—C14	1.405 (3)	H31—C18	1.232 (16)
C15—H28	1.159 (10)	H32—C18	1.09 (3)
C16—F2	1.347 (4)	H33—C18	1.12 (3)
C16—C12	1.393 (2)	H34—C22	1.113 (7)
C16—C13	1.397 (3)	H35—N10	1.301 (15)
C17—N7	1.359 (4)	H36—C23	1.167 (7)
C17—N9	1.317 (3)	H37—C24	1.170 (9)
C17—H30	1.121 (13)	H38—C26	1.14 (4)
C18—N7	1.462 (3)	H39—C26	1.14 (3)
C18—H31	1.232 (16)	H40—C27	1.14 (3)
C18—H32	1.09 (3)	H41—C27	1.14 (3)
C18—H33	1.12 (3)	H42—O6^ii^	0.85 (3)
			
N10—O4—C26	109.6 (3)	N7—C18—H33	113.1 (19)
C27—O6—H42^i^	100 (3)	H31—C18—H33	104.3 (16)
C11—N7—C17	105.96 (9)	H32—C18—H33	114.8 (12)
C11—N7—C18	125.63 (16)	O5—C19—N10	122.8 (3)
C17—N7—C18	126.3 (3)	O5—C19—C14	119.2 (3)
C13—N8—C20	125.0 (4)	N10—C19—C14	114.1 (4)
C13—N8—H29	124.7 (9)	N8—C20—C21	119.9 (3)
C20—N8—H29	110.4 (9)	N8—C20—C22	122.5 (4)
C12—N9—C17	103.2 (2)	C21—C20—C22	117.4 (2)
O4—N10—C19	119.8 (3)	F3—C21—C20	118.0 (2)
O4—N10—H35	120.0 (10)	F3—C21—C23	117.6 (3)
C19—N10—H35	114.3 (9)	C20—C21—C23	123.8 (2)
N7—C11—C12	105.26 (15)	C20—C22—C24	119.3 (6)
N7—C11—C15	132.8 (2)	C20—C22—H34	122.8 (8)
C12—C11—C15	121.7 (3)	C24—C22—H34	117.1 (8)
N9—C12—C11	110.31 (13)	C21—C23—C25	117.3 (4)
N9—C12—C16	132.0 (3)	C21—C23—H36	122.4 (5)
C11—C12—C16	117.59 (18)	C25—C23—H36	120.1 (4)
N8—C13—C14	119.8 (3)	C22—C24—C25	119.0 (5)
N8—C13—C16	122.2 (3)	C22—C24—H37	122.4 (6)
C14—C13—C16	117.8 (2)	C25—C24—H37	118.1 (5)
C13—C14—C15	120.4 (3)	Br1—C25—C23	117.8 (2)
C13—C14—C19	120.22 (19)	Br1—C25—C24	120.1 (3)
C15—C14—C19	117.4 (5)	C23—C25—C24	122.07 (18)
C11—C15—C14	119.0 (3)	O4—C26—C27	109.1 (9)
C11—C15—H28	124.7 (7)	O4—C26—H38	110 (3)
C14—C15—H28	116.2 (7)	C27—C26—H38	110 (2)
F2—C16—C12	118.6 (2)	O4—C26—H39	109 (3)
F2—C16—C13	118.3 (4)	C27—C26—H39	110 (3)
C12—C16—C13	122.8 (2)	H38—C26—H39	109.5 (17)
N7—C17—N9	113.8 (2)	O6—C27—C26	114.1 (11)
N7—C17—H30	119.5 (10)	O6—C27—H40	107 (3)
N9—C17—H30	125.4 (9)	C26—C27—H40	109.4 (19)
N7—C18—H31	107.9 (7)	O6—C27—H41	109 (2)
N7—C18—H32	109.4 (14)	C26—C27—H41	109 (2)
H31—C18—H32	107 (2)	H40—C27—H41	108.6 (15)

Symmetry codes: (i) *x*−1/2, −*y*+3/2, −*z*+1; (ii) *x*+1/2, −*y*+3/2, −*z*+1.

### (binimetinib_VASP) Crystal data

**Table:**

C_17_H_15_BrF_2_N_4_O_3_	*b* = 16.01200 Å
*M**_r_* = 441.23	*c* = 4.86608 Å
Orthorhombic, *P*2_1_2_1_2_1_	*V* = 1804.71 Å^3^
*a* = 23.16240 Å	*Z* = 4

### (binimetinib_VASP) Data collection

**Table:**

*h* = →	*l* = →
*k* = →	

### (binimetinib_VASP) Fractional atomic coordinates and isotropic or equivalent isotropic displacement parameters (Å^2^)

**Table:**

	*x*	*y*	*z*	*B*_iso_*/*B*_eq_	
Br1	0.21583	1.19671	0.75423		
F2	0.17429	0.77297	0.66775		
F3	0.16697	0.90877	0.32040		
O4	−0.10831	0.89653	0.26129		
O5	−0.04005	0.85262	0.68214		
O6	−0.21696	0.91024	0.54783		
N7	0.09310	0.60081	0.01736		
N8	0.07664	0.87294	0.71560		
N9	0.17259	0.63256	0.26145		
N10	−0.05892	0.84888	0.22432		
C11	0.07868	0.67001	0.17370		
C12	0.12943	0.68901	0.32456		
C13	0.07819	0.80469	0.53543		
C14	0.02728	0.78152	0.38710		
C15	0.02719	0.71394	0.20461		
C16	0.12781	0.75578	0.50880		
C17	0.14903	0.58161	0.07714		
C18	0.05533	0.55671	−0.17077		
C19	−0.02660	0.82946	0.44334		
C20	0.10956	0.94556	0.71049		
C21	0.15473	0.96344	0.52698		
C22	0.09601	1.00905	0.90051		
C23	0.18688	1.03650	0.53834		
C24	0.12630	1.08382	0.91226		
C25	0.17252	1.09668	0.73321		
C26	−0.15852	0.84578	0.19082		
C27	−0.21257	0.89367	0.26039		
H28	−0.01202	0.69634	0.09477		
H29	0.03603	0.88130	0.79338		
H30	0.17098	0.52878	−0.01938		
H31	0.01541	0.53825	−0.06465		
H32	0.07718	0.50010	−0.24257		
H33	0.04410	0.59684	−0.34606		
H34	0.06091	0.99782	1.04600		
H35	−0.04755	0.83869	0.02052		
H36	0.22171	1.04588	0.39098		
H37	0.11482	1.13114	1.06363		
H38	−0.15639	0.78740	0.30960		
H39	−0.15748	0.83253	−0.03098		
H40	−0.21384	0.95254	0.14206		
H41	−0.24900	0.85504	0.18852		
H42	−0.25621	0.89297	0.61096		

### (binimetinib_VASP) Hydrogen-bond geometry (Å, º)

**Table:**

*D*—H···*A*	*D*—H	H···*A*	*D*···*A*	*D*—H···*A*
N8—H29···O5	1.02	1.90	2.727	136
N10—H35···O5^i^	1.04	1.67	2.675	161
O6—H42···N9^ii^	1.00	1.81	2.806	176
C17—H30···O6^iii^	1.09	2.18	3.221	158
C18—H32···O5^iii^	1.10	2.53	3.288	125
C26—H38···Br1^iv^	1.10	2.92	3.841	142
C26—H39···Br1^iii^	1.10	2.78	3.486	122

Symmetry codes: (i) *x*, *y*, *z*−1; (ii) *x*−1/2, −*y*+3/2, −*z*+1; (iii) −*x*, *y*−1/2, −*z*+1/2; (iv) −*x*, *y*−1/2, −*z*+3/2.
